# Metabolomic characterisation of the effects of oncogenic *PIK3CA* transformation in a breast epithelial cell line

**DOI:** 10.1038/srep46079

**Published:** 2017-04-10

**Authors:** Chung-Ho E. Lau, Gregory D. Tredwell, James K. Ellis, Eric W.-F. Lam, Hector C. Keun

**Affiliations:** 1 Division of Cancer, Department of Surgery and Cancer, Imperial College London, Hammersmith Hospital, London, UK; 2 Research School of Physics and Engineering, Department of Applied Mathematics, Australian National University, Canberra, Australia

## Abstract

Somatic mutations in *PIK3CA* are frequently found in a number of human cancers, including breast cancer, altering cellular physiology and tumour sensitivity to chemotherapy. This renders *PIK3CA* an attractive molecular target for early detection and personalised therapy. Using ^1^H Nuclear Magnetic Resonance spectroscopy (NMR) and Gas Chromatography – Mass Spectrometery (GC-MS) together with ^13^C stable isotope-labelled glucose and glutamine as metabolic tracers, we probed the phenotypic changes in metabolism following a single copy knock-in of mutant *PIK3CA* (H1047R) in the MCF10A cell line, an important cell model for studying oncogenic transformation in breast tissues. We observed effects in several metabolic pathways, including a decrease in glycerophosphocholine level together with increases in glutaminolysis, *de novo* fatty acid synthesis and pyruvate entry into the tricarboxylic acid cycle. Our findings highlight altered glyceroplipid metabolism and lipogenesis, as key metabolic phenotypes of mutant *PIK3CA* transformation that are recapitulated in the MCF10A cellular model.

*PIK3CA* encodes for the 110 kDa p110α subunit of the class 1 phosphatidylinositol 3-kinase (PI3K), a family of lipid kinases that are involved in regulating molecular growth and survival signalling. Second only to TP53, *PIK3CA* is one of the most frequently mutated genes in breast tumours, and a comprehensive study recently found that as many as 36% of all breast tumours harboured *PIK3CA* mutations^1^,^2^. It has been suggested that *PIK3CA* mutation may have prognostic value in predicting survival outcome in breast cancer patients^3^ – partly because *PIK3CA* mutations tend to be associated with hormone receptor-positive tumours that are responsive to hormone therapies^4^. Somatic *PIK3CA* mutations observed in tumours are oncogenic and result in increased catalytic PI3K kinase activity. PI3K phosphorylates phosphatidylinositol 4,5-diphosphate (PIP_2_) and produces phosphatidylinositol 3,4,5-triphosphate (PIP_3_), a lipid second messenger that activates the PI3K-AKT signalling cascade^5^. The PI3K-AKT signalling pathway is important in cancer cells, as it is associated with many hallmarks of cancer, such as enhanced cell proliferation, genomic instability, angiogenesis and inflammatory response^6^,^7^. Consequently, as part of the drive towards targeted therapies, a number of small chemical inhibitors were developed to target the activity of PI3K signalling pathway at various nodes, and several are currently in clinical trials. However, early results from these clinical trials generally showed limited single agent activity in advanced tumours^8^,^9^. This is partly because PI3K pathway inhibition can lead to the selection for compensatory pathways which restore survival and tumour growth^10^. More effective treatments are currently required to help target the *PIK3CA*-mutant patient populations - one strategy that has been suggested is to exploit tumour metabolic dependency^11^,^12^ by discriminating the metabolic regulation of the various oncogenic mutations. AKT signalling has been reported to stimulate glucose metabolism^13^, and mutant *PIK3CA* has also been shown to increase growth dependence on glucose^14^ and glutamine^15^.

MCF10A is a spontaneously immortalized non-tumorigenic mammary epithelial cell line derived from a 36-year old patient, and it displays many characteristics of normal breast epithelium^16^. It is a valuable model for studying disease progression, epithelial-mesenchymal transition and metabolism^17^,^18^,^19^. By performing a series of metabolomics experiments in the non-transformed MCF10A mammary epithelial line, here we report the metabolic alterations resulting from a single copy knock-in of mutant *PIK3CA (H1047R*). We identified increased glutaminolysis, *de novo* fatty acid synthesis, pyruvate entry into the TCA cycle, and decreased glycerophosphocholine as the most prominent phenotypes following *PIK3CA* mutation.

## Materials and Methods

MCF10A and *PIK3CA H1047R* (+/−) mutant MCF10A cells were purchased from Horizon Discovery Ltd (HD, Cambridgeshire, United Kingdom).

### Cell Culture

MCF10A cells were cultured in DMEM/F12, supplemented with 5% horse serum, 0.1 μg/ml cholera toxin, 20 ng/ml hEGF, 10 μg/ml insulin, 0.5 μg/ml hydrocortisone, and 2 mM L-glutamine. Cells were cultured as a monolayer at 37 °C in a humidified atmosphere with 5% CO_2_ under normal oxygen conditions. The cells were passaged every 3–4 days, and were split 1:8–1:12 at ~80% confluency. Only low passage cells, less than 5 passages from the state when these were purchased were used for experiments.

### Proliferation assay

The number of viable cells in individual wells of 96-well plates was determined using the colorimetric cell counting kit-8 (CCK8) following the manufacturer’s instructions (Sigma-Aldrich). 10 μl of the CCK8 reagent were added to wells in 96 well plates and incubated for 3 hours; the absorbance at 450 nM was then measured for each well and was subtracted from background.

### ^13^C-glucose and ^13^C-glutamine labelling experiment

On the day of seeding: after trypsinisation, MCF10A cells were resuspended in full media (DMEM/F12, supplemented with 5% horse serum, 0.1 μg/ml cholera toxin, 20 ng/ml hEGF, 10 μg/ml insulin, 0.5 μg/ml hydrocortisone, 2 mM L-glutamine) and 120 × 10^3^ cells were seeded per well on a six well plate and were allowed to adhere overnight. The medium was aspirated the next day (24 hours after seeding) and was replaced with experimental culture media, and incubated for 24 hours. The media used for the glucose-labelled experiment were as follows: glucose free, glutamine free, pyruvate free DMEM, supplemented with 10% dialysed-FBS (BioSera), and 11.2 mM U-^13^C_6_-glucose and 2 mM L-glutamine. The media used for the glutamine-labelled experiment were as follow: glucose-free, glutamine-free, pyruvate-free DMEM, supplemented with 10% dialysed-FBS (BioSera), 11.2 mM glucose and 2 mM ^13^C_5_-glutamine. Dialysed serum was used to filter out the serum small molecule metabolite background to ensure content consistency in the experiment. Three independent biological replicate experiments were performed with U-^13^C_6_ glucose, and four independent biological replicate experiments were performed with U-^13^C_5_ glutamine.

The samples were harvested after 24 hours. The media were collected and immediately placed on ice. The cell monolayer was washed with 500 μL of cold (4* °*C) Ringer’s buffer, which was aspirated before the addition of 750 μL of cold methanol (straight from a −20* °*C freezer and kept cold in ethanol bath). The methanol-quenched cells were then scraped from the surface of the well and the entire sample was transferred to a clean 2 ml eppendorf tube. To increase metabolite recovery, each well was washed with a further 750 μL of cold methanol and pooled with the first sample. The methanol-quenched samples were dried down in a rotary evaporator under reduced pressure. Representative wells from each cell line*/*condition were used for cell counting at the beginning and at the end of the experiments; cell counting was done using a Sceptor^TM^ 2.0 Cell Counter (Millipore). For the extracellular media samples 1 ml of the culture media were transferred to fresh Eppendorf™ tubes, and were centrifuged (8000 × g, 5 min) to remove potential cell debris. They were then stored at −80* *°C for subsequent analysis. Both dried down cell samples and media were stored in an −80* *°C freezer.

### Intracellular Metabolite extraction for metabolomics analysis

A dual-phase methanol/chloroform method was then used to separate out the aqueous metabolites, and the non-polar metabolites from the cell proteins. Metabolites were extracted from the dried down methanol-quenched cell pellet samples and the samples were kept on ice during the extraction. 300 μl of chloroform/methanol in a 2:1 ratio was added to the cell pellet and was mixed using vortex. Then 300 μl of HPLC/UPLC graded H_2_O was added to the samples, which was again mixed using vortex and centrifuged at 16000 g for 5 min.

### Sample preparation of culture medium for ^1^H NMR analysis

Culture medium samples were kept frozen in eppendorfs in −80 °C freezers after sample harvesting, and samples were prepared into NMR tubes on the day of the spectroscopic analysis. After the samples were thawed, they were kept on ice. 550 μl of the medium sample was transferred into a new eppendorf, and 50 μl of internal standard DSA (4,4-Dimethyl-4-silapentane-1-ammonium trifluoroacetate) in D_2_O (11.6 mM) was added into the sample as a quantitation reference. The mixture was then pipetted into a standard 5 mm NMR tube.

### Sample preparation of intracellular metabolites for ^1^H NMR analysis

After methanol/chloroform/water dual phase extraction, aqueous fractions were dried down in eppendorfs using a freeze-dryer. The dried down samples were then reconstituted in 600 μl of phosphate buffer (composition of the phosphate buffer: 0.2 M Na_2_HPO_4_, 0.043M NaH_2_PO_4_, 100 μM TSP, 3 mM NaN_3_ in 100% D_2_O). Samples were then centrifuged at 16,000 g for 5 mins to spin down any insoluble material, and 550 μl of the reconstituted samples were transferred to clean standard 5 mm NMR tubes.

### ^1^H NMR experiment acquisition and data processing

High-resolution ^1^H NMR spectra were acquired using either a 5 mm broadband-inverse tube probehead or a 5-mm cryoprobe using a 14.1T Bruker AVANCE 600 spectrometer (Bruker Biospin). Carr- Purcell-Meilboom-Gill (CPMG) spectra were acquired at 300 K using a standard presat pulse sequence, with the fixed echo time (τ) set at 400 μs and the total spin echo time of 64 ms. Spectra were recorded with 64 transient scans, following 16 dummy scans. A 3 s relaxation delay was incorporated, and gradient shimming was used before all spectral acquisitions to improve magnetic field homogeneity across the detected sample volume. Cell Media Data were imported and processed in MATLAB^®^ (MathWorks) using scripts written in house by J.T. Pearce, H.C. Keun, T.M.D. Ebbels, and R. Cavill (Imperial College London, UK). ^1^H NMR spectra were automatically phased, baseline-corrected, and referenced to the internal standard resonance at 0 ppm. Spectral integration was performed in MATLAB^®^ (MathWorks) after metabolite identification. Identifying metabolites from the signal peaks are made often through the use of databases at the Human Metabolome Database and at the Biological Magnetic Resonance Bank, or through published literature. Spectral fitting of intracellular levels of glycerophosphocholine, phosphocholine and choline were performed using ChenomxNMR suite Profiler (version 8.1, Chenomx Inc, Edmonton, Canada).

### Sample preparation of intracellular aqueous metabolites for GC-MS analysis

10 μl of 1.5 mg/ml myristic acid-d27 was added to the dried aqueous fractions as an internal standard, and the samples were dried down using a vacuum concentrator (SpeedVac^TM^). Samples were first derivatised through methoxyamination, where 20 μl of methoxyamine (20 mg/ml in anhydrous pyridine) was added to samples using a multipipette, and samples were mixed using vortex and spun in centrifuge. The samples were placed in a heater block for 90 min at 37 °C. At the end of the period, samples were cooled and spun in a centrifuge again. Samples were then silylated by adding 80 μl of MTBSTFA (with 1% TBDMS) (Thermo). After mixing by vortex, and centrifugation, samples were placed in a heater block, and were incubated for a further 60 min at 70 °C. At the end of the period, samples were cooled and spun in a centrifuge again. Finally 10 μl of 1 mM 2-fluorobiphenyl (in anhydrous pyridine) was added to the samples as an injection standard, and the samples were then transferred to deactivated glass vial inserts.

### Sample preparation of non-polar metabolites for GC-MS analysis

10 μl of 1.5 mg/ml myristic acid-d27 was added to the samples as an internal standard. The samples were dried down using a vacuum concentrator before they were reconstituted in 333 μl of methanol/toluene solution (1:1 v/v ratio), and were treated with 167 μl of 0.5 M sodium methoxide and incubated at room temperature for 1 hour. Reaction was halted by the addition of 500 μl of 1 M NaCl and 25 μl of concentrated HCl. The fatty acids were then extracted using two volumes of hexane (500 μl), and the combined organic layers were dried under N_2_. Samples were then reconstituted with 40 μl acetonitrile, silylated by adding 40 μl of MSTFA (with 1% TMCS) (Thermo), and were incubated for 30 min at 37 °C. At the end, 10 μl of 1 mM injection standard 2-fluorobiphenyl (in anhydrous pyridine) was added to the samples.

### GC-MS instrument set up and data processing

GC-MS analysis was performed on an Agilent 7890 GC system connected to an Agilent 5975 MSD triple-axis detector operating under electron impact ionization (Agilent Technologies). Metabolites were separated with a 30 m DB-5MS capillary column with an attached 10 m Duraguard column. Samples were injected with an Agilent 7693 autosampler injector into deactivated splitless using helium as the carrier gas. The analysis was performed based on the Fiehn method^20^ and the data were acquired under selected ion monitoring (SIM) mode, with representative samples from each biological group also run under full scan mode. The identities of the GC-MS features were confirmed either through running standards or matching to the NIST library, aided by an in-house generated library using the AMDIS program for deconvolution^21^. Individual isotopomer peaks were integrated using in-house MATLAB^®^ (MathWorks) scripts by Dr G. D. Tredwell (Imperial College London) based on the program GAVIN^22^. The mass isotopomer distribution vectors (MID) for each metabolite were normalised i.e. the sum of the metabolite isotopomer abundances equal to one. MATLAB^®^ (MathWorks) scripts were written in-house by Dr G. D. Tredwell (Imperial College London) to automatically correct for naturally occurring elemental isotopes based on the method described by Millard *et al*.^23^.

### Isotopomer Spectral Analysis (ISA)

ISA was performed with MATLAB^®^ scripts developed in-house by Dr G. D. Tredwell (Imperial College London)^24^. The computation provided estimates for two (D, G) or three (D, G, E) parameter ISA models based on minimising the differences between the acquired spectral mass isotopomer distribution data and the data simulated using method described in the method section.

### Statistical analysis

Principal component analysis and one-way ANOVA were performed in MATLAB^®^. Student’s t-tests were computed either in MATLAB^®^ or Microsoft Excel.

## Results

To investigate the metabolic alterations resulting from the single copy knock-in of mutant *PIK3CA (H1047R*) in MCF10A cells, stable isotope tracer and metabolomics experiments were performed on heterozygous *H1047R* mutant cells, and results were compared to the isogenic parental line expressing wild type PIK3CA. Unlabelled glucose or glutamine was substituted with either uniformly ^13^C-labelled glucose or uniformly ^13^C-labelled glutamine, to enable discrimination of the fate of these two major nutrients. While MCF10A is spontaneously immortalized, growth factors such as EGF and insulin are normally used in routine cell culture to stimulate growth. However, both are critical modulators of multiple signalling pathways and hence it would be desirable to observe their metabolic phenotypes with the additives removed. Thus, prior to the metabolic profiling, experiments on the impact of media change, from fully supplemented to without supplements, on growth phenotype in the wild type and mutant *PIK3CA* cells were assessed using a proliferation assay. Reduced growth factor availability did not affect the mutant *PIK3CA* cells. However, significant differences in growth were observed in the wild type cells by 48 hours (Figure S1). Hence, 24 hours was selected as the time period over which subsequent metabolomics experiments were conducted, and cell numbers at the beginning and at the end of the metabolomics experiment were measured (Figure S2).

### Mutant *PIK3CA* decreases pyruvate production and increases glutamate release in MCF10A cells

**Figure 1:**
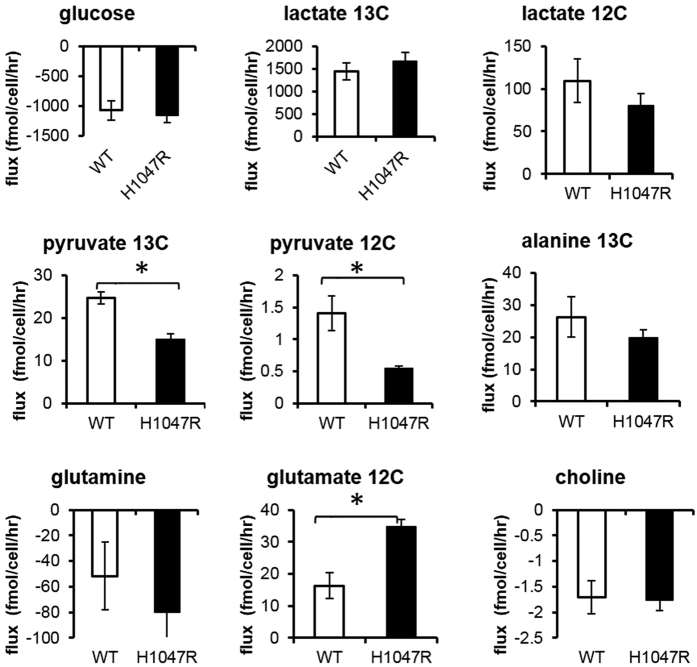
MCF10A metabolite consumption and release from culture medium. Samples from U-^13^C_6_ glucose tracer experiment harvested after 24 hours were analysed by ^1^H NMR. Negative values indicate consumption and positive values indicate net efflux; detailed resonance assignments can be found in the supplementary section. Bar graphs represent mean ± SEM from three independent biological replicates. *Denotes Student’s t-test p values < 0.05.

In culture, metabolite concentrations in the extracellular environment could trigger metabolic feedback^25^,^26^ and regulate important functional phenotypes^27^,^28^, and are thus valuable physiological indicators. We employed ^1^H NMR spectroscopy to analyse the spent culture media from the U-^13^C-glucose labelling experiment, which enabled us to examine the consumption and release profiles of key metabolites, including the uptake of glucose, glutamine and choline, and the production of pyruvate, lactate, and glutamate (Fig. 1, Tables S3 and S4). We noticed that extracellular pyruvate release was significantly decreased by ~40% (p = 0.01) in the *PIK3CA* mutant cells compared to the parental wild type cells. Pyruvate was predominantly an intermediate metabolite of glucose metabolism, with over 90% of the methyl carbon of pyruvate found to be glucose-derived in this cell model (Fig. 1). However, despite a decrease in pyruvate release in the *PIK3CA* mutant cells, both glucose consumption and glucose-derived lactate release remained unaffected, suggesting that any alteration of the fate of pyruvate was limited to either the fraction entering mitochondria or pyruvate converted to alanine. Furthermore, glutamate release and glutamine uptake were also affected in the *PIK3CA* mutant cells. Non-glucose-derived glutamate release, as assessed by the C4 glutamate proton resonance, doubled (*p* = 0.02), while glutamine uptake recorded an approximately 50% increase (pairwise t-test *p* value < 0.05) in the *PIK3CA* mutant cells compared to the wild type parental line, signifying up-regulation of glutamine utilisation and metabolism. Glutamine, once imported into the cells is converted to αKG via glutamate to replenish substrates in the TCA cycle, and glutaminolysis has been reported to support growth and survival in rapidly proliferating tumour cells^29^. Overall, our data on extracellular pyruvate, glutamine and glutamate suggest that metabolic substrate entry into the TCA cycle might have been altered in the transformed *PIK3CA* mutant cells. In addition, we were also able to measure uptake of extracellular choline in the culture media, and found no significant differences between the *PIK3CA* mutant and the wild type MCF10A parental cells.

### Intracellular aqueous metabolites analysis: pyruvate entry into TCA cycle is altered in the *PIK3CA* transformed cells

**Figure 2:**
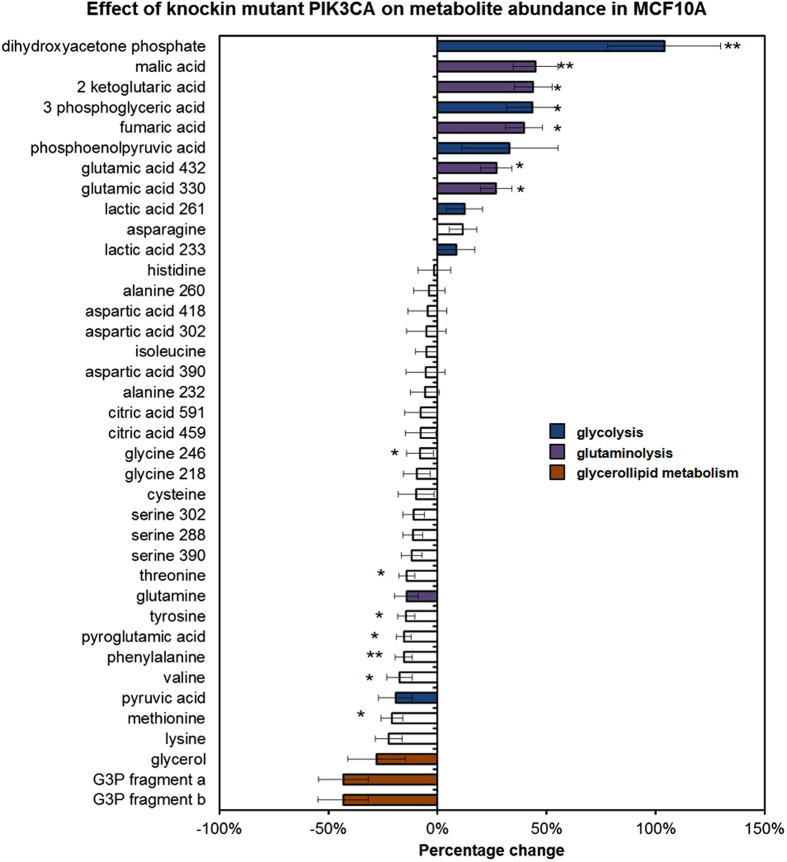
*PIK3CA* mutation altered intracellular aqueous metabolite abundance in MCF10A cells. Analysis was performed by GC-MS and data from both U-^13^C_6_ glucose and U-^13^C_5_ glutamine tracer experiments are included. For each metabolite the ion signals from individual isotopomers were summed and the metabolite intensity dataset were normalised through median fold change normalisation^55^. The bar graphs represent the mean ± SEM. Seven separate biological experiments are represented and the metabolite features are ranked according to the magnitude of difference between the mutant *PIK3CA* and the wild type parental line, with positive change representing an increase in the mutant compared to wild type cells. Multiple mass ion fragments may be detected for some metabolites; in those cases the m/z values of the distinct fragments are given e.g. lactate 261 (see Table S1). G3P (glycerol-3 phosphate) fragments are eluted at two separate retention times; *pairwise Student’s t-test p values < 0.05; **pairwise Student’s t-test p values < 0.005.

**Figure 3:**
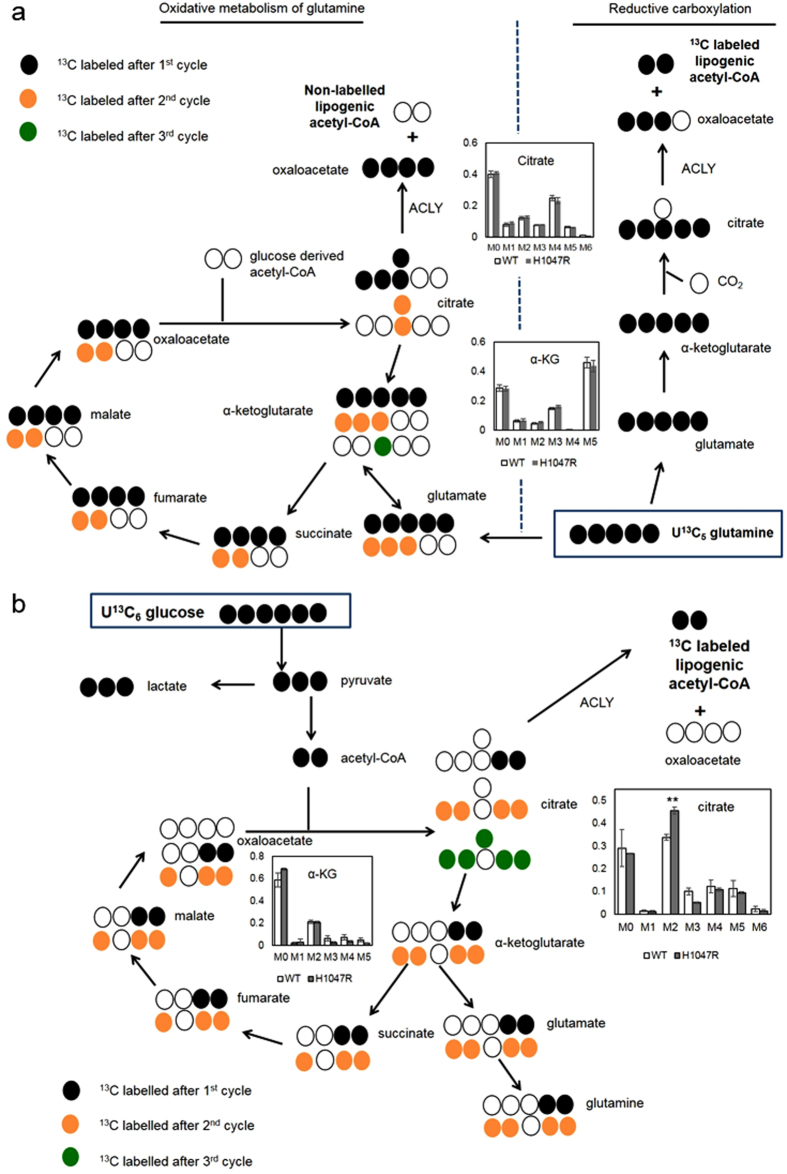
Schematic diagram of central carbon and tricarboxylic acid cycle metabolism. ^13^C glucose and glutamine tracers labeling schematic and comparison of mass isotopomer distribution (MID) of citrate and 2-ketoglutaric acid (αKG) from ^13^C glucose and glutamine tracers. In the glucose tracer data (**a**), the bar graphs represent the mean ± SEM from three separate biological replicate experiments in the glucose tracer data. In the glutamine tracer data (**b**), the bar graphs represent the mean ± SEM from four separate biological replicate experiments. **Student’s t-test p < 0.005; *Student’s t-test p < 0.05.

GC-MS profiling of the intracellular metabolites from the U-^13^C_6_ glucose and U-^13^C_5_ glutamine-labelled cultures enabled us to systematically compare the relative abundance of metabolites between the *PIK3CA* mutant and the non-transformed cells, to determine the ^13^C mass isotopomer distribution, and to apportion the overall molecular carbons derived from glucose and glutamine for each metabolite. When examining the relative abundance data, we summed up the raw intensities of individual mass isotopomers of each aqueous metabolites and found the relative quantities of glycolytic intermediates (dihydroxyacetone phosphate, PEP, 3PG) and TCA cycle intermediates (αKG, fumarate, malate, glutamate) to be consistently higher (p < 0.05) in the mutant cell samples (Fig. 2, Table S1), possibly indicating that the metabolic demand for energy generation is higher in the *PIK3CA*-transformed cells. At the same time we found glutamine to be an important metabolic precursor to TCA cycle intermediates. While glucose and glutamine each contributed around equally to the citrate carbon skeleton (around 30% each), glutamine accounted for over 40% of the malate and fumarate carbons versus less than 30% derived from glucose (Figures S3 and S4). Together with increased pool size in metabolites associated with glutaminolysis (glutamate, αKG, fumarate, malate), these data indicate that glutamine is a more important carbon donor than glucose in maintaining TCA cycle intermediate pools in mutant MCF10A cells. Also, we observed that the carbon flow was predominantly in the oxidative direction of the TCA cycle as opposed to reductive carboxylation – as evident by the higher level of glutamine derived citrate labelled M4 compared to citrate labelled M5 (Fig. 3a). Whilst all five carbons from U-^13^C_5_ glutamine are incorporated into M5 citrate isotopomers under reductive carboxylation, M4 labelled citrate would be derived after U-^13^C_5_ glutamine has undergone one turn of the TCA cycle. Increased extracellular glutamine uptake and glutamate production could be used to fuel higher TCA activities in the mutant *PIK3CA* MCF10A cells. Furthermore, by closely inspecting the mass isotopomer distributions of individual metabolites we detected sizeable and significant shifts in the means by which ^13^C glucose carbons were incorporated into citrate (Fig. 3b). In particular, whilst the overall citrate pool remained unaltered in this model the citrate M2 label derived from U-^13^C_6_ glucose tracer increased by around one third (*p* < 0.005), whereas citrate M3 label decreased by one half (*p* < 0.05) in the *PIK3CA* mutant cells. The synthesis of citrate is normally catalysed by citrate synthase, which utilises four-carbon oxaloacetate and two-carbon acetyl-CoA as substrates. Mitochondrial acetyl-CoA is predominately derived from pyruvate via pyruvate dehydrogenase activity whereas oxaloacetate can be formed by carboxylation of pyruvate or oxidation of malate. Importantly, pyruvate dehydrogenase contributes to citrate M2 labels (^13^C_6_ glucose 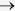
^13^C_3_ pyruvate 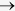
^13^C_2_ acetyl-CoA 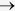
^13^C_2_ citrate) and pyruvate carboxylase contributes to citrate M3 labels (^13^C_6_ glucose 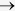
^13^C_3_ pyruvate 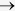
^13^C_3_ oxaloacetate 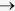
^13^C_3_ citrate). The increase in M2 citrate labels accompanied by the decrease in M3 citrate labels indicated that pyruvate entry into citrate via pyruvate dehydrogenase was increased relative to pyruvate carboxylase flux in the *PIK3CA* mutant MCF10A cells.

### Analysis of lipid species: increased de novo lipid synthesis in the *PIK3CA* mutant MCF10A

**Figure 4:**
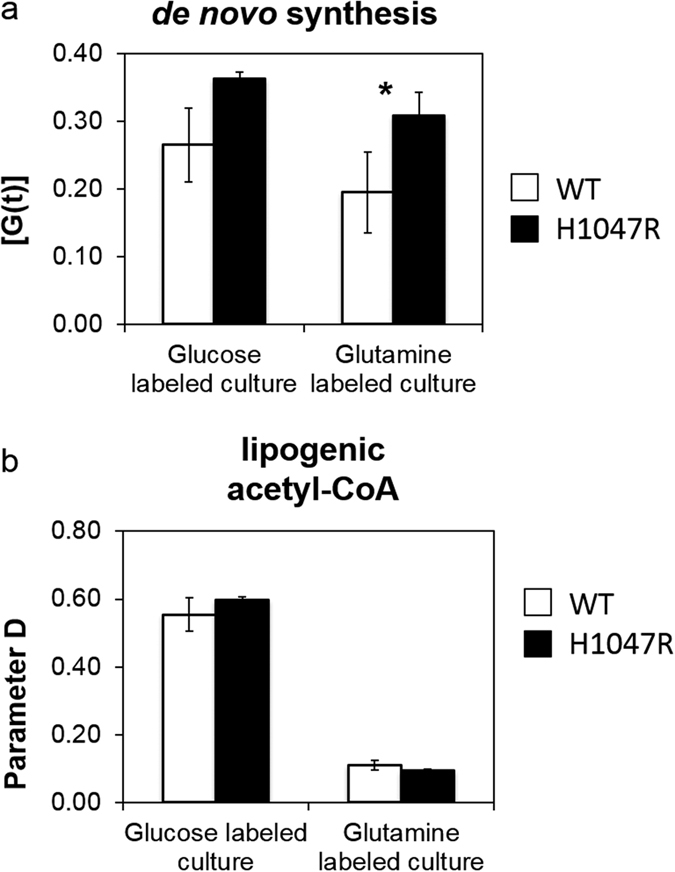
Modelled metabolic parameters from fatty acid Isotopomer Spectral Analysis (ISA). ISA parameters were modelled based on mass isotopomer distribution (MID) of methyl palmitate. In the glutamine tracer data, the graphs represent the mean ± SEM from four independent biological replicate experiments and in the glucose tracer data the graphs represent the mean ± SEM data from three independent biological replicate experiments. Two-tailed Student’s t-test was used to determine statistical significance, and *denotes t-test p < 0.05.

Intracellular lipids were analysed by GC-MS using an extraction and derivatisation method that enabled both fatty acid esters and free fatty acids to be detected. The fatty acid methyl esters detected were not restricted to a specific class of lipid molecule, but are fatty acid chains from all lipid molecules, which were transesterified in the dervitisation process. These transesterified fatty acids could comprise various lipids, such as; membrane phospholipids (phosphatidylcholine) or signalling and functional lipids (phosphatidic acid or diacylglycerol). Our analysis successfully measured various lipid bound fatty acids, and we were able to examine the relative abundance of a number of methyl esters (lipid bound fatty acids) and the free fatty acid oleate (Table S2). For example, we found that both ratios of esterified linolenate to palmitate and esterified linolenate to oleate were significantly lower (pairwise t-test p < 0.05) in the *PIK3CA* mutant extracts. Whilst oleate and palmitate can be synthesised *de novo*, linolenate (C18:3) is an essential polyunsaturated fatty acid in mammalian cells and must be imported from the culture medium directly; our data indicated there was a possible shift in the *PIK3CA* mutant cells, away from relying on fatty acid uptake and towards *de novo* biosynthesis. Furthermore, the mass isotopomer data from U-^13^C_6_ glucose and U-^13^C_5_ glutamine both provided strong independent evidence that the rate of *de novo* biosynthesis of fatty acids was elevated in the *PIK3CA* mutant cells. In particular, we found increased incorporation of both glucose and glutamine derived two-carbon acetyl-CoA units into methyl palmitate (Figures S5 and S6), the most abundant fatty acid chain in mammalian cells. By modelling the mass isotopomer distribution of methyl palmitate using Isotopomer Spectral Analysis (ISA), a technique that untangles the effect of changes in the acetyl CoA pool contribution from the biosynthetic rate^24^, it was established that the increases in ^13^C tracer label incorporation were results of higher rates of *de novo* biosynthesis in the *PIK3CA* mutant cells (Fig. 4, Table S6). Upon culture with U-^13^C_5_ glutamine the *PIK3CA* mutant cells demonstrated higher *de novo* biosynthesis of palmitate (lipid bound methyl palmitate was 50% higher (*p* < 0.05) when compared to the wild type MCF10A parental line). Additionally, the modelled data suggest that glucose-derived citrate was preferentially used as substrate for forming lipogenic acetyl-CoA; roughly 60% of acetyl-CoA came from glucose as opposed to around 10% from glutamine (Fig. 4).

### Analysis of glycerophosphocholine metabolism

**Figure 5:**
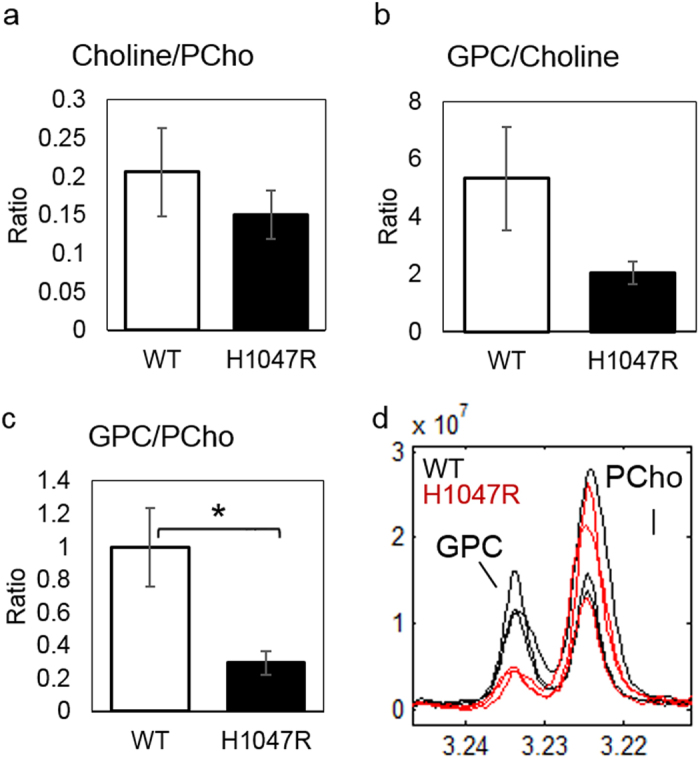
Analysis of intracellular choline, phosphocholine (PCho) and glycerophosphocholine (GPC) by ^1^H NMR. The bar graphs (**a–c**) represent the mean ± SEM from three separate biological replicate experiments. Details of resonance assignment can be found in Table S5. Two-tailed Student’s t-test was used to determine statistical significance and *represents p < 0.05. (**d**) NMR spectrum of PCho and GPC signals in the wild type and the mutant samples.

During the analysis of the intracellular aqueous metabolites, we noted a substantial drop of approximately 40% (not reaching significance) in the relative abundance of [glycerol-3 phosphate (G3P) + glycerophosphocholine (GPC)] in the *PIK3CA* mutant cells, ranking it the most down-regulated metabolite feature in this study (Fig. 2). The mass fragment (m/z: 591) represents the primary ion fragment from the derivatisation of G3P and the ambiguity of the assignment is due to the fact that the choline moiety in GPC can spontaneously detach under high temperature leaving the remaining molecule to be derivatised as G3P. To resolve this ambiguity additional GC-MS analyses of G3P and GPC standards were performed to find additional mass fragments that discriminated between the two metabolites. We were able to identify a distinct GPC fragment peak (m/z: 325) and a putative structure for the fragment (Figure S7). Furthermore by referring back to the original sample data acquired under full scan mode, we found remarkable similarity in the patterns of U-^13^C_6_ glucose mass isotopomer distributions between GPC and the ambiguously assigned [G3P + GPC] ion fragments (Figure S7), suggesting that GPC contributed substantially to the [G3P + GPC (m/z: 591)] fragment signals in the dataset. The U-^13^C_6_ glucose mass isotopomer distribution data also showed that GPC M3 levels were 38% lower in the *PIK3CA* mutant cells (*p* < 0.05, Figure S8), indicating that the turnover of GPC and its glucose-derived glycerol carbon backbone were lower in the *PIK3CA* mutant MCF10A. To further verify these observations, the level of GPC was also quantified using solution state ^1^H NMR and 70% reductions was observed in the GPC: PCho (*p* < 0.05) ratio (Fig. 5, Table S5).

## Disscussion

MCF10A is an immortalised, non-transformed cell line retaining many features of normal breast epithelium^16^, and is widely used to study the phenotypic changes of oncogenic transformations. The signalling pathway modulation induced by a single copy knock-in of mutant *PIK3CA* (H1047R) in the MCF10A cells have previously been examined, and it was reported that the three main recurrent somatic *PIK3CA* hotspot mutations (H1047R/E542K/E545K) all promote constitutive AKT and ERK activation in MCF10A, leading to growth factor independent growth^30^,^31^. To our knowledge, the most comprehensive study available to date examining the global impact of this mutant in the MFC10A cells is currently provided by Hart *et al*.^32^, whom have characterised the transcriptome, proteome, and metabolome of two cell lines. Our current study provides further evidence of changes in metabolic phenotypes in the MCF10A cells resulting from the single knock-in *PIK3CA (H1047R*) mutation by utilising ^13^C stable isotope-labelled glucose and glutamine as tracers. We consider our study to be complementary to the data presented in Hart *et al*.

### *PIK3CA* transformation is associated with metabolic reprogramming in MCF10A

**Figure 6:**
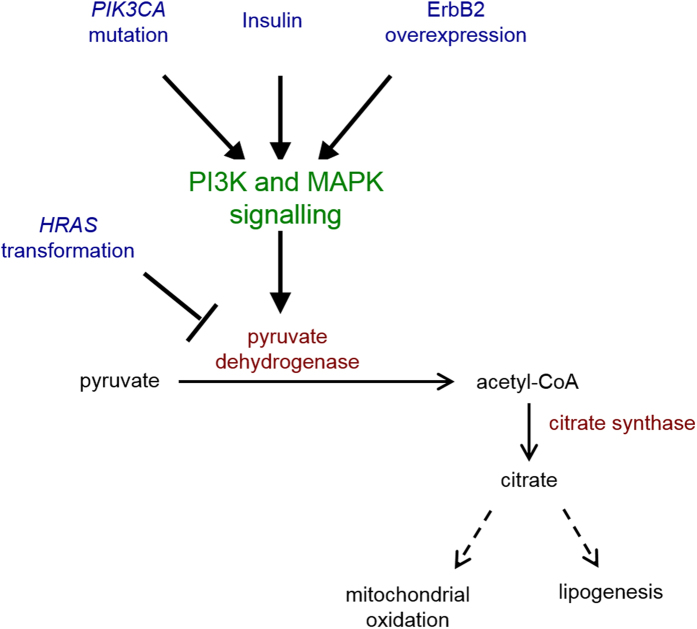
Pyruvate dehydrogenase regulation and oncogenic transformation in MCF10A.

We found evidence that *PIK3CA* mutant transformation in MCF10A cells modulated pyruvate metabolism. In particular cells with mutant *PIK3CA* exhibited reduced pyruvate efflux into the culture medium and increased pyruvate conversion to acetyl-CoA, helping to fuel TCA cycle activity. Our data suggests pyruvate dehydrogenase activity may be modulated as a result of the mutation. Pyruvate dehydrogenase can be inhibited via post-transcriptional modification by pyruvate dehydrogenase kinases (PDKs). In transcriptomic data presented in Hart *et al*. (Figure S9) describing the same cell model, pyruvate dehydrogenase kinase isoforms *PDK2* and *PDK3* mRNA levels were consistently lower in the *PIK3CA* mutant cells, consistent with up-regulation of pyruvate dehydrogenase activity. Pyruvate dehydrogenase activity is known to be modulated through multiple mechanisms, including insulin^33^, MAPK^34^ and PI3K signalling pathways. While elevated pyruvate dehydrogenase flux, resulting from down-regulation of PDK4^35^, has been reported in ErbB2-overexpressing MCF10A, it was found to be down-regulated in HRAS transfected MCF10A cells^36^, illustrating that regulation of pyruvate dehydrogenase activity is oncogene and mutation specific in the MCF10A cells (Fig. 6). It is possible that mutant *PIK3CA* supports higher pyruvate flux into TCA cycle through pathways involving both PI3K/AKT and MAPK/ERK signalling.

We also found evidence of increased glutamine uptake and glutamate production in the PIK3CA transformed cells. It has been reported that mutant PIK3CA enhances ATP generation in MCF10A cells^18^, and the additional energy supply could be met through increased mitochondrial oxidation and glutaminolysis, a process which is normally under the transcriptional control of c-myc^37^. PTEN is a phosphatase which acts to reverse PI3K activity, and has been shown to repress c-myc and glutaminolysis in mice^38^. Interestingly, we also noted both magnitude and significance of increase in glutamate production were greater than in glutamine consumption. It is possible glutamate production could be additionally affected by changes in glutamate transporter (SLC7A11/SLC1A3) activity. The cystine/glutamate antiporter (SLC7A11) couples the export of glutamate with cystine import - critical to glutathione production and has been shown to play an important role in modulating glutamate secretion in breast cell culture^39^. However SLC7A11 peptide levels were significantly decreased in PIK3CA mutant cells, and only the aspartate-linked glutamate transporter (SLC1A3) increased at both the mRNA and protein expression level. Thus the cause of this phenotype remains unclear.

We showed that mutant *PIK3CA* stimulated *de novo* fatty acid biosynthesis in MCF10A cells. Proliferating transformed cells can meet higher biomass demand either by scavenging, as has been reported for oncogenic *KRAS* transformed cells^40^, or through increased *de novo* synthesis. ATP citrate lyase has been reported to be a direct target of AKT^41^ and is required for generating lipogenic acetyl-CoA. Interestingly, according to data presented in Hart *et al*. (Figure S10) the protein levels in all three enzymes key to fatty acid synthesis - ATP citrate lyase (ACLY) acetyl-CoA carboxylase (ACACA) and fatty acid synthase (FASN) were also higher in *PIK3CA* mutant MCF10A cells.

### Regulation of glycerophosphocholine metabolism

A decrease in the glycerophosphocholine to phosphocholine ratio (GPC:PC) has previously been reported to be associated with disease progression and immortalization in mammary epithelial cells^42^. Here we observed evidence of a decrease in GPC:PC which appeared to be mostly attributable to a decrease in GPC specifically following *PIK3CA* transformation. PI3K inhibitor studies using ^1^H NMR spectroscopy have also reported an alteration in the glycerophosphocholine and phosphocholine phenotype, typically a decrease in phosphocholine^43^,^44^. Previous studies have mainly attributed phosphocholine levels to changes in choline uptake^45^ and choline kinase expression^43^,^46^, which converts choline to phosphocholine. However, less is known about the regulation of glycerophosphocholine. Interestingly, in this study the apparent changes in glycerophosphocholine appeared to be independent of choline uptake capacity, and are unlikely to be accounted for by the differences in cell growth alone (Figure S2). The origin of changes in the glycerophosphocholine phenotype remains unclear, but it has been suggested that reduced glycerophosphocholine turnover following oncogenic transformation could be indicative of lower phosphatidylcholine degradation upon enhanced survival signalling^47^,^48^. EDI3/GPCPD1 is responsible for the recycling of glycerophosphocholine, and according to data presented in Hart *et al*. (Figure S9) the transcript level for *GPCPD1* is higher in MCF10A *PIK3CA* mutant cells which supports the role of this enzyme in producing the glycerophosphocholine phenotype observed in the mutant model. EDI3/GPCPD1 regulates migration and invasion of MCF7 breast cancer cells and is a significant prognostic factor in endometrial cancer^49^. This disease is also characterized by a high frequency of mutations in the PI3K/AKT pathway^50^, hinting at possible links between *PIK3CA* mutation, glycerophosphocholine recycling and metastatic phenotypes in several tumour types. However in a previous study of intact human endometrial tumour using magic angle spinning NMR spectroscopy, we did not observe a clear decrease in GPC levels despite significant upregulation of *GPCPD1* gene expression^45^. Other NMR studies of breast cancer suggest that, rather than correlating with aggressive disease, lower GPC and GPC:PC may be associated with better outcomes in some subtypes of tumour^51^,^52^. The relationship between *PIK3CA* mutation and prognosis is also dependent on breast cancer subtype^2^,^53^, and can be due to a modulation of therapeutic response as well as instrinsic tumour biology. Clearly additional studies will be required to understand the significance of GPC metabolism, its relationship to PI3K/AKT signaling and malignancy.

### Limitations and Future work

MCF10A cells have distinct signalling and genetic backgrounds to breast tumour cell lines^16^ which could interfere with the knock-in *PIK3CA* mutation^54^. It is possible that the effect of knock-in *PIK3CA* mutation on metabolic regulation we observed might be to some extent dependent on specific genetic background and context. Employing additional cell lines as alternative models, such as *PTEN*-deleted models would help identify and address any confounding effects from genetic and signalling interactions.

In this study, we described a series of metabolic alterations following a single copy knock-in *PIK3CA (H1047R*) mutation in MCF10A breast cells, which included de novo fatty acid synthesis, pyruvate entry into mitochondria, and depletion of glycerophosphocholine. Our data suggest the *PIK3CA (H1047R*) mutation led to increased fatty acid synthesis in the MCF10A cells and it would be of value to further characterise its lipidome in detail and also measure the levels of intracellular lipid droplets in the two cell lines. It would be of interest to ascertain if *PIK3CA* mutation-induced metabolic alterations would be reversible, through the use of PI3K/AKT inhibitors and activators.

When we compared the relative metabolite abundance data in our study to that presented in Hart *et al*.^32^, we have noted some discrepancies particularly in the level of amino acids observed (Fig. 2 and Figure S11). These may be due to metabolic exchange and feedback resulting from differences in the culture medium composition used in the two studies.

## Conclusions

This study demonstrates that prominent metabolic phenotypes associated with oncogenic *PIK3CA* transformation in MCF10A cells include increased pyruvate entry into mitochondrial citrate and enhanced *de novo* fatty acid synthesis. We also observed evidence that *PIK3CA* mutation induces glycerophosphocholine depletion. These findings confirm that this model recapitulates some of the metabolic effects associated with activation of PI3K/AKT signaling, and highlights the potential of metabolomics to detect biomarkers of oncogenic transformation.

## Additional Information

**How to cite this article**: Lau, C.-H. E. *et al*. Metabolomic characterisation of the effects of oncogenic *PIK3CA* transformation in a breast epithelial cell line. *Sci. Rep.* **7**, 46079; doi: 10.1038/srep46079 (2017).

**Publisher's note:** Springer Nature remains neutral with regard to jurisdictional claims in published maps and institutional affiliations.

## Supplementary Material

Supplementary Information
