# Supplementary material for: Metabolomic characterisation of the effects of oncogenic PIK3CA transformation in a breast epithelial cell line
Source: Sci Rep. 2017 Apr 10;7:46079. doi: 10.1038/srep46079 (PMC5385542; doi:10.1038/srep46079)
Supplement: Supplementary Information [file srep46079-s1.pdf]

SUPPLEMENTARY INFORMATION

Metabolomic characterisation of the effects of oncogenic PIK3CA transformation in a breast epithelial cell line

Chung-Ho E. Lau[1], Gregory D. Tredwell[2], James K. Ellis[1], Eric W-F Lam[1], Hector C. Keun[1]

[1]Division of Cancer, Department of Surgery and Cancer, Imperial College London, Hammersmith Hospital, London, UK.

[2]Research School of Physics and Engineering, Department of Applied Mathematics, Australian National University, Canberra, Australia

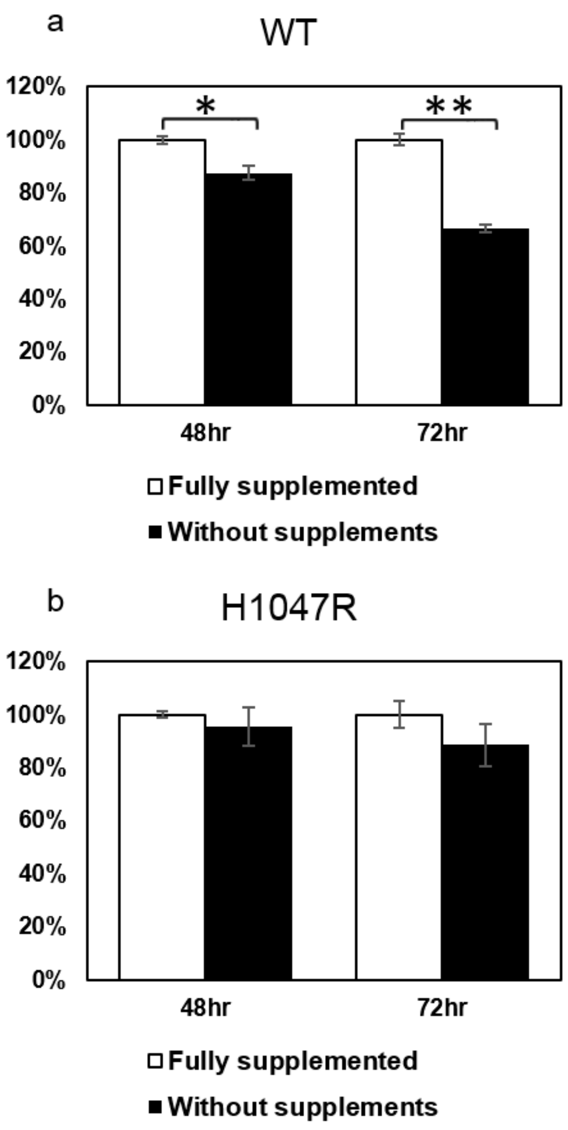

Figure S1. Effect of withdrawing supplements on growth and proliferation. Effect of withdrawing supplements on MCF10A wild type (A) and mutant *PIK3CA* cells (B) over 48 hours and 72 hours. Fully supplemented cells were provided with EGF, insulin, hydrocortisone and cholera toxin and relative cell growth was assessed using the colorimetric CCK8 proliferation assay performed in a 96 well plate format. Cells were plated at low density and in excess volume of culture medium, and no media change took place over the 72 hours. Data represent the relative growth compared to cells cultured in fully supplemented media at the specified timepoint, and data from the two cell lines were independently normalised. The bar graphs represent the mean ± SEM from four technical replicates. Two-tailed

Student's t tests were performed to evaluate the statistical significance of growth under these two conditions.

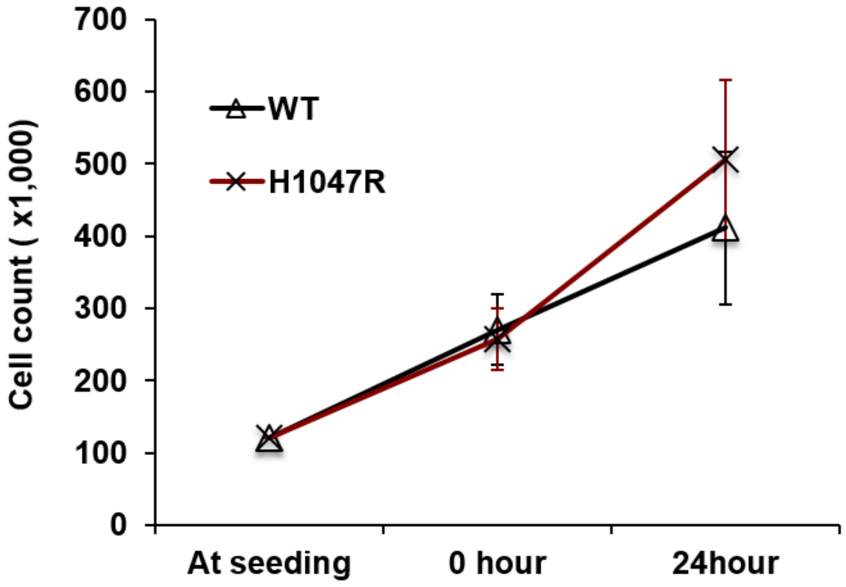

Figure S2. Growth in wild type MCF10A and *PIK3CA* mutant MCF10A cells. Cell count data of the MCF10A wild type and mutant *PIK3CA* cells over the course of the ^{13}C-glucose and ^{13}C-glutamine labelling experiments. Cell counting was performed using an automated cell counter, and cells were seeded on a 6 well plate 24 hours before they were replenished with ^{13}C labelled tracer-containing media. The graphs represent the mean ± SEM from a total of 7 replicates. The difference in cell number at 24 hours was not found to be statistically significant using two-tailed Student's t test.

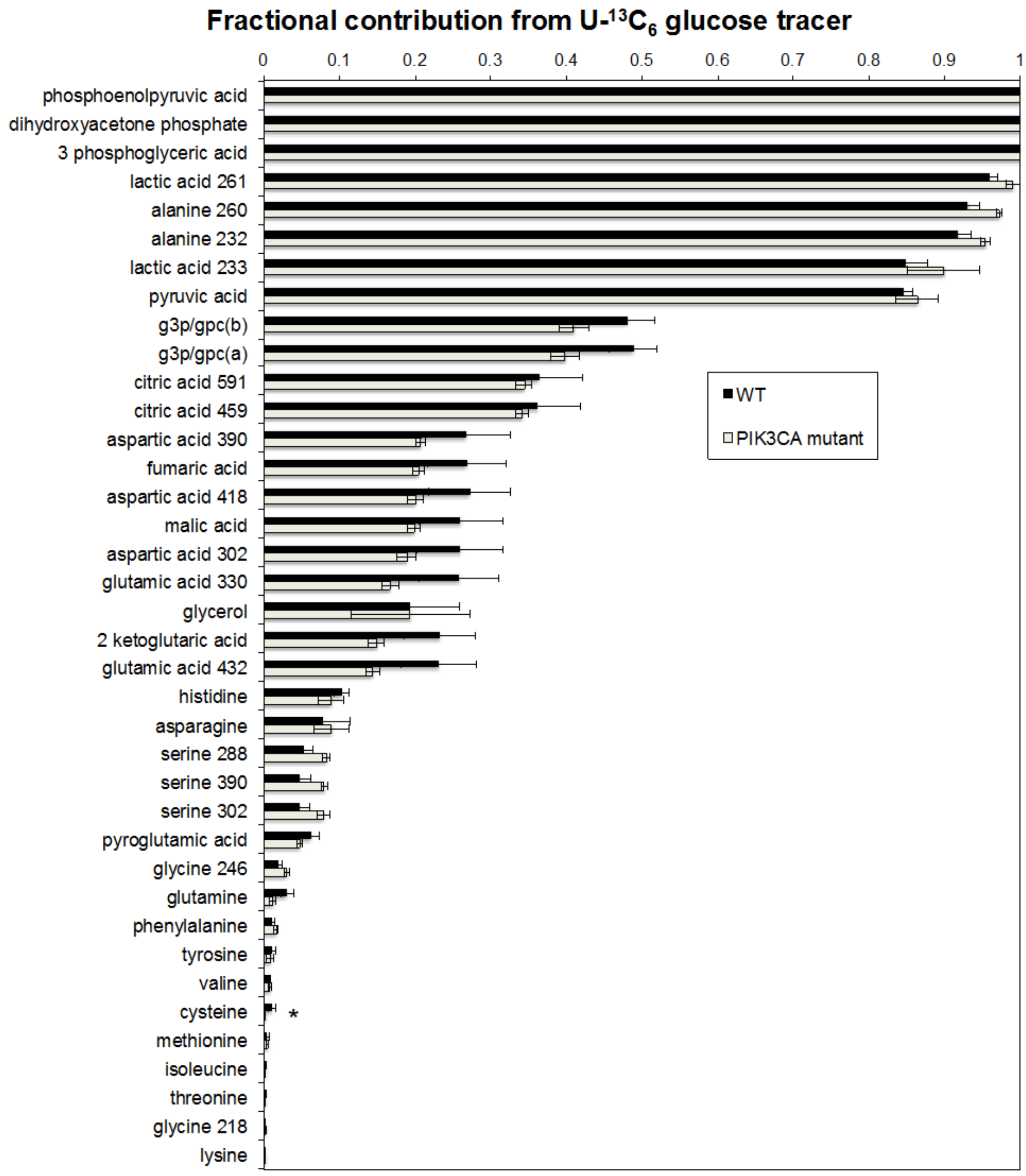

Figure S3. ^{13}C glucose carbon incorporation into aqueous metabolites. Incorporation is calculated using the natural abundance-corrected mass isotopomer distribution (MID) data from the U-$^{13}C_6$ glucose tracer cultured samples. The bar graphs represent the mean ± SEM from three separate biological replicate experiments. * two-tailed Student's t-test $p < 0.05$. Multiple mass ion fragments may be detected for some metabolites; in those cases the m/z values of the distinct fragments are given e.g. lactate 261 (see Table S). GPC+G3P was eluted at two separate retention times.

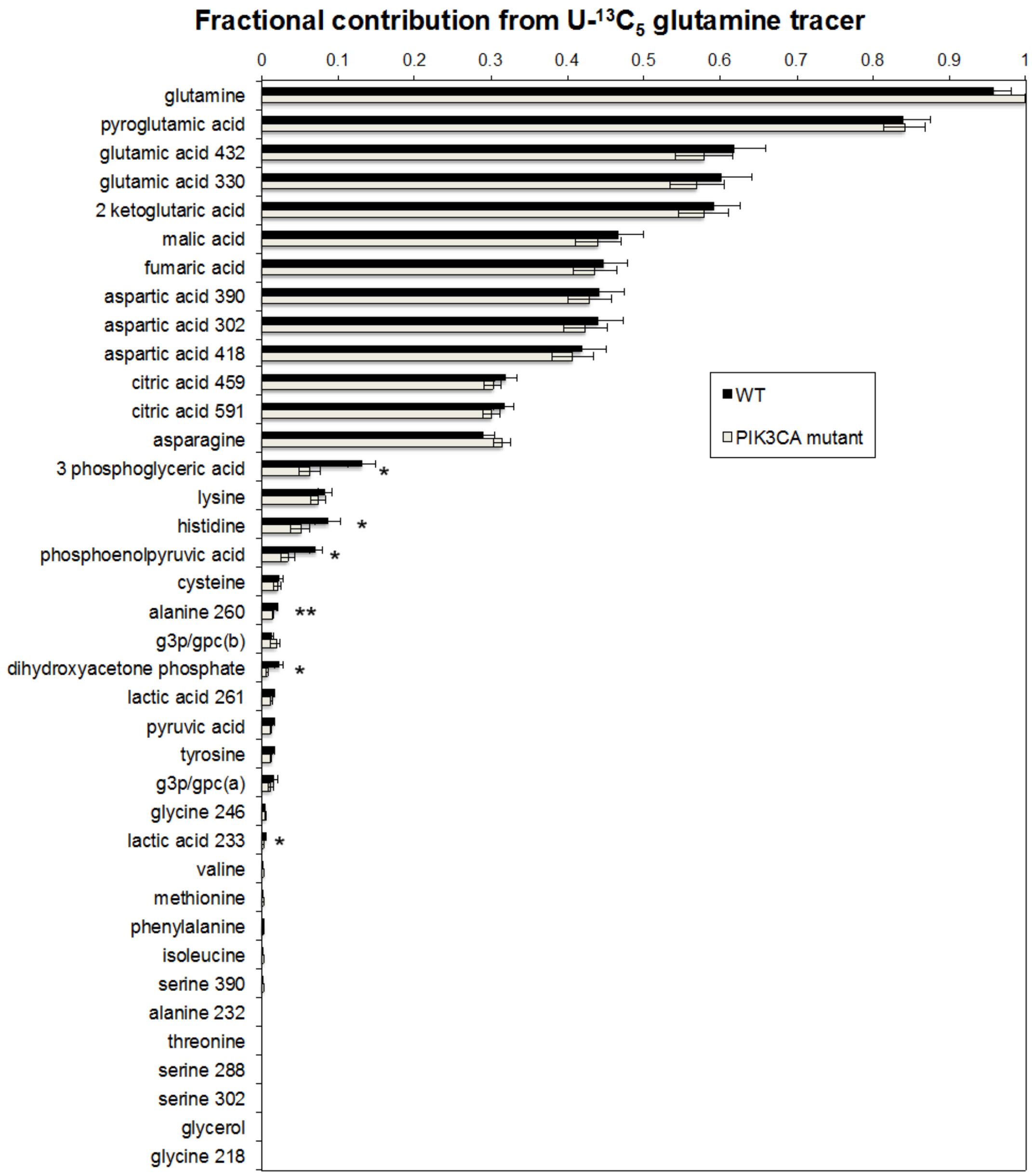

Figure S4. ^{13}C glutamine carbon incorporation into aqueous metabolites. Incorporation is calculated using the natural abundance-corrected mass isotopomer distribution (MID) data from the U-$^{13}C_5$ glutamine tracer experiments. The bar graphs represent the mean ± SEM from four separate biological replicate experiments. * two-tailed Student's t-test $p < 0.05$; ** two-tailed Student's t-test $p < 0.005$. Multiple mass ion fragments may be detected for some metabolites; in those cases the m/z values of the distinct fragments are given e.g. lactate 261 (see Table S). Glycerol-3 phosphate+ glycerophosphocholine (G3P+GPC) was eluted at two separate retention times.

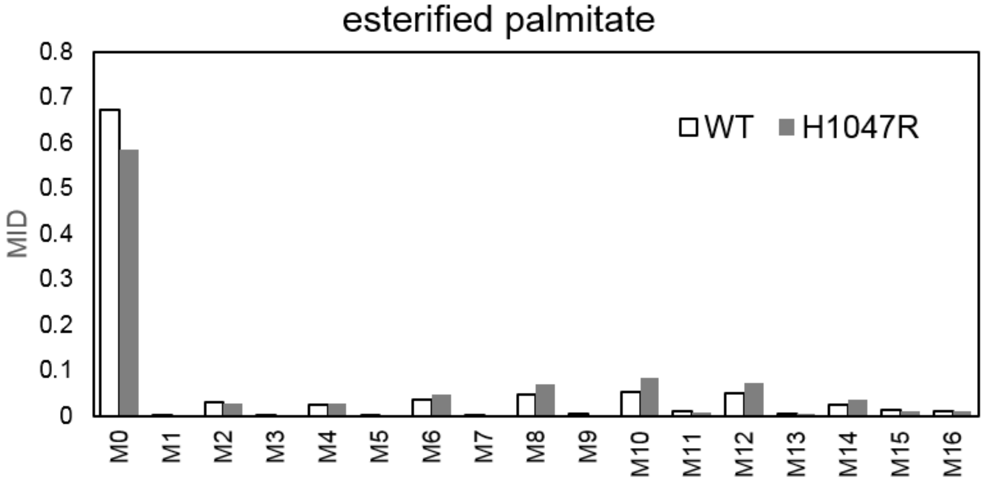

Figure S5. U-$^{13}C_6$ glucose carbon incorporation into transesterified palmitate. The bar graphs represent the mean ± SEM from three separate biological experiments.

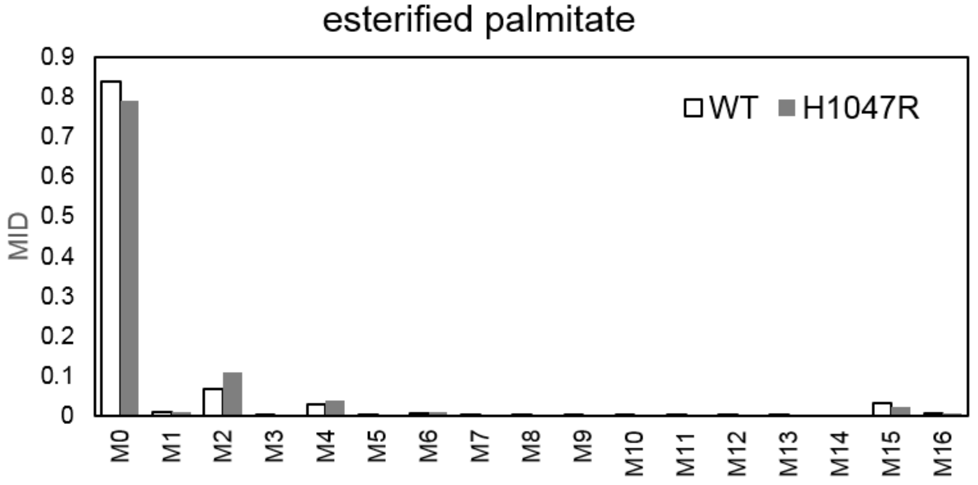

Figure S6. U-$^{13}C_5$ glutamine carbon incorporation into transesterified palmitate. The bar graphs represent the mean ± SEM from four independent biological experiments.

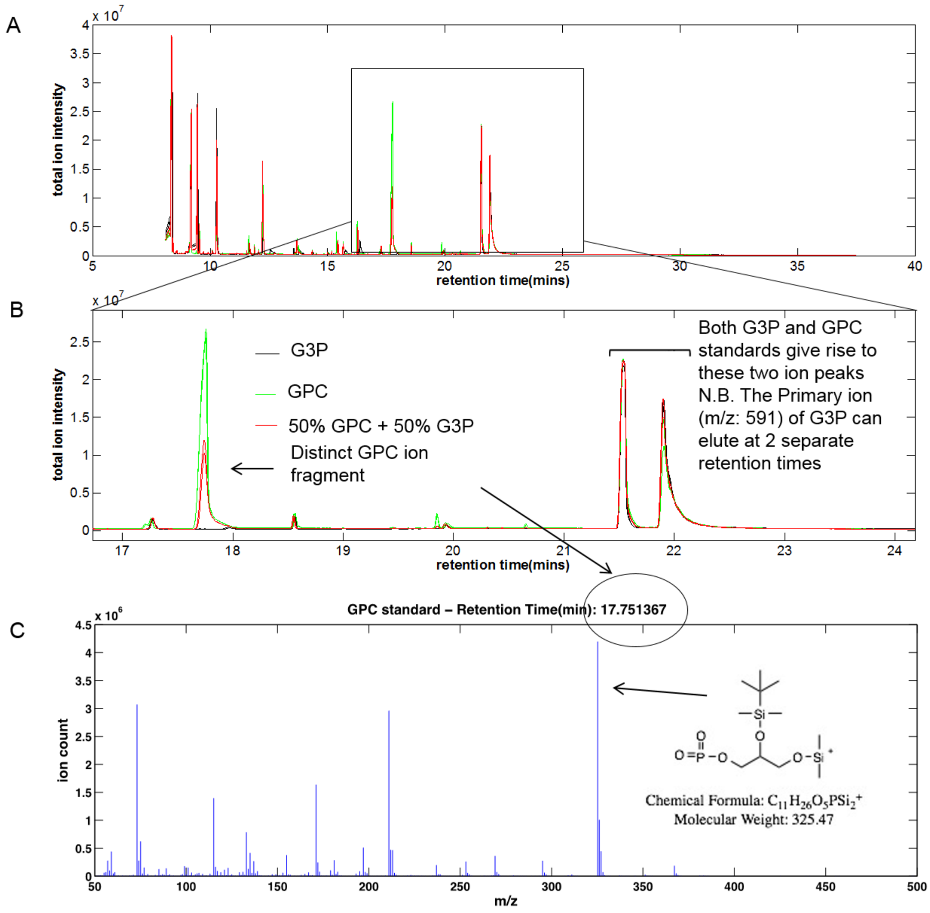

Figure S7. GC-MS Assignment of glycerophosphocholine (GPC) fragment through standard runs. Standards of GPC (green), G3P (black) were made up to 1mg/ml and, additionally a standard of 50:50 GPC:G3P mix (red) from the 1mg/ml stocks was also made up. Standards were run under identical protocols to the MCF10A cell samples. (A) The full GC-MS total ion chromatogram. (B) Enlarged version of the GC-MS total ion chromatogram. (C) Mass spectrum at RT 17.75mins from the GPC standard sample showing the dominant ion fragment (m/z 325) and its putative structure.

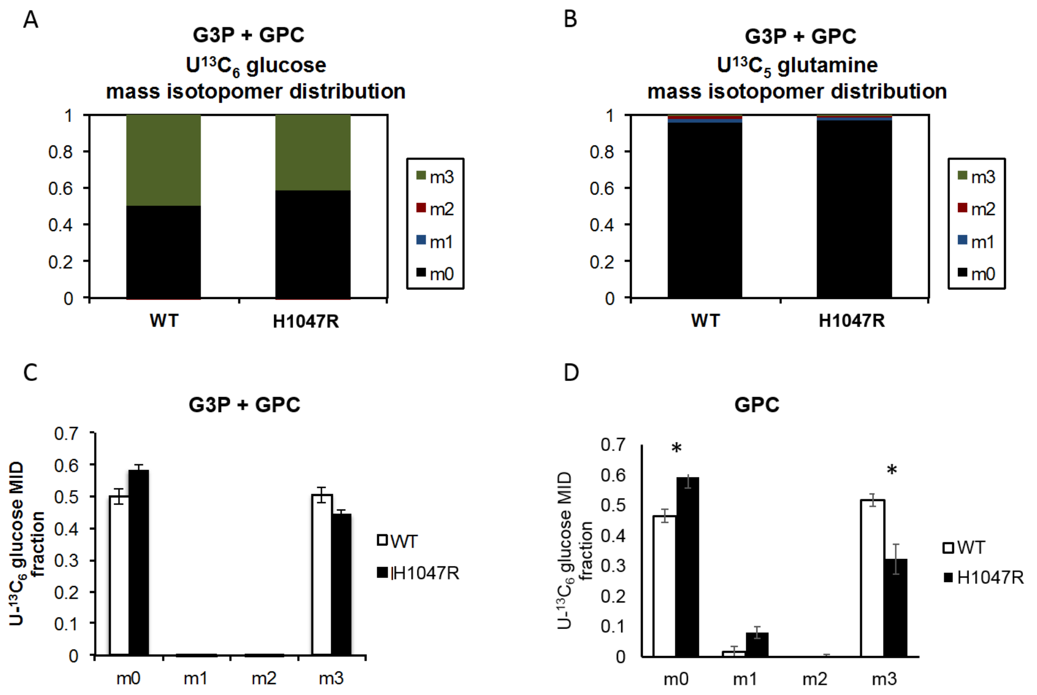

Figure S8. The glycerol carbon backbone of glycerophosphocholine is derived primarily from glucose, but not glutamine. Alteration in glycerophosphocholine (GPC) glucose carbon mass isotopomer distribution could be responsible for the changes observed in the overall G3P+GPC pool. The bar graphs represent the mean ± SEM from three separate biological replicate experiments. * represents two-tailed Student's t-test $p < 0.05$. Measurements of GPC m1 ion (m/z: 326) were affected by background signals which can be observed in the blank sample.

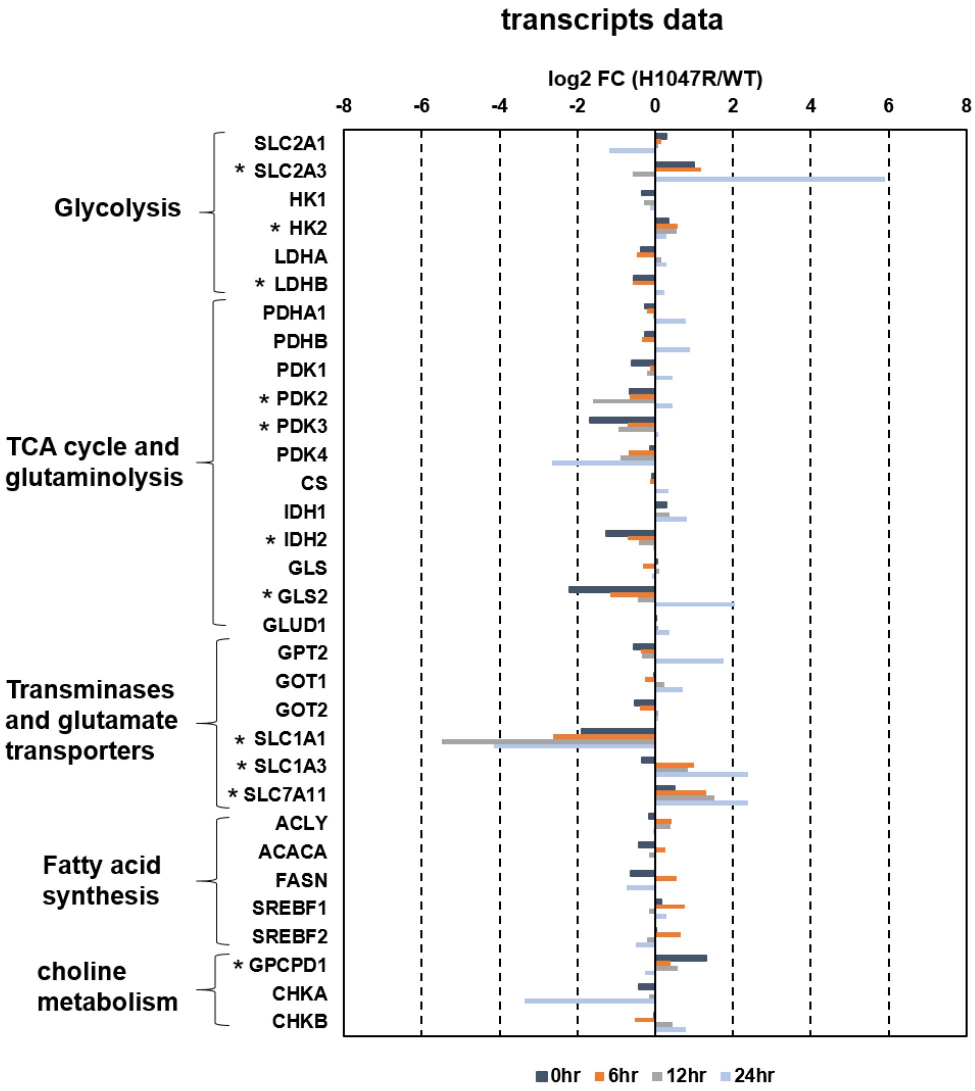

Figure S9. Comparisons of key metabolic gene transcripts levels based on available literature data. The data represent log 2 fold changes in key metabolic genes comparing *PIK3CA H1047R* cells to wild type MFC10A cells at 0, 6, 12 and 24 hours following media change; a positive fold change represents gene transcript was found higher in the mutant cells compared to wild type. * $p < 0.005$ in at least 2 of the 4 timepoints. This analysis is based on processed RNAseq data obtained from - *The butterfly effect in cancer: A single base mutation can remodel the cell* [1].

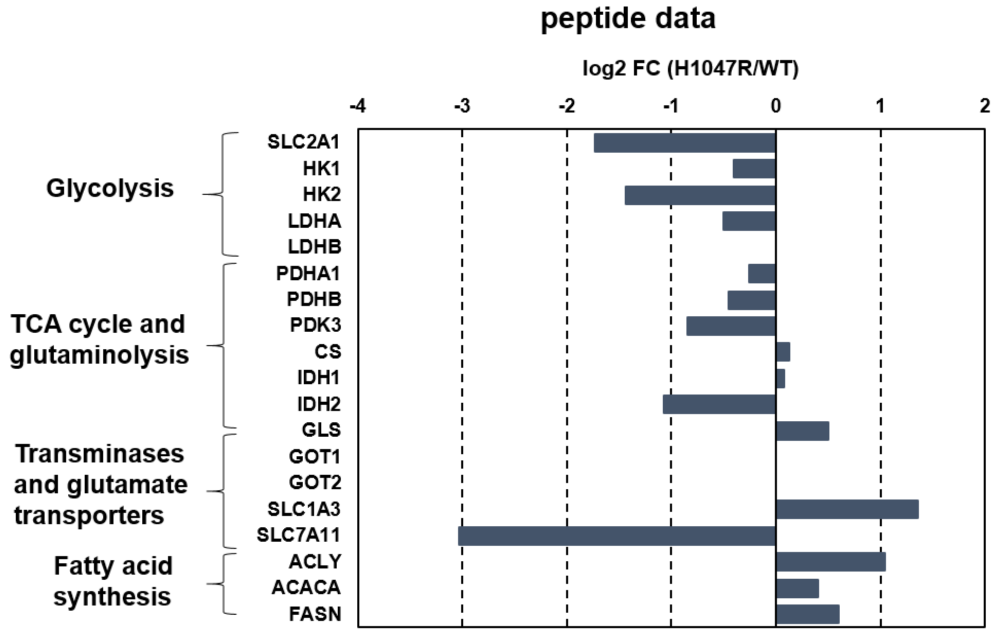

Figure S10. Comparisons of key metabolic proteins based on available literature data. The data represent log 2 fold changes in protein peptide levels comparing *PIK3CA H1047R* cells to wild type MFC10A cells; a positive fold change represents peptide was found in higher abundance in the mutant cells compared to wild type. All peptides shown have $p < 0.001$ except HK1, LDHB, CS, IDH1, GOT1 and GOT2. This analysis is based on SILAC (stable isotope labelling by amino acids in cell culture) data first presented in *The butterfly effect in cancer: A single base mutation can remodel the cell* [1].

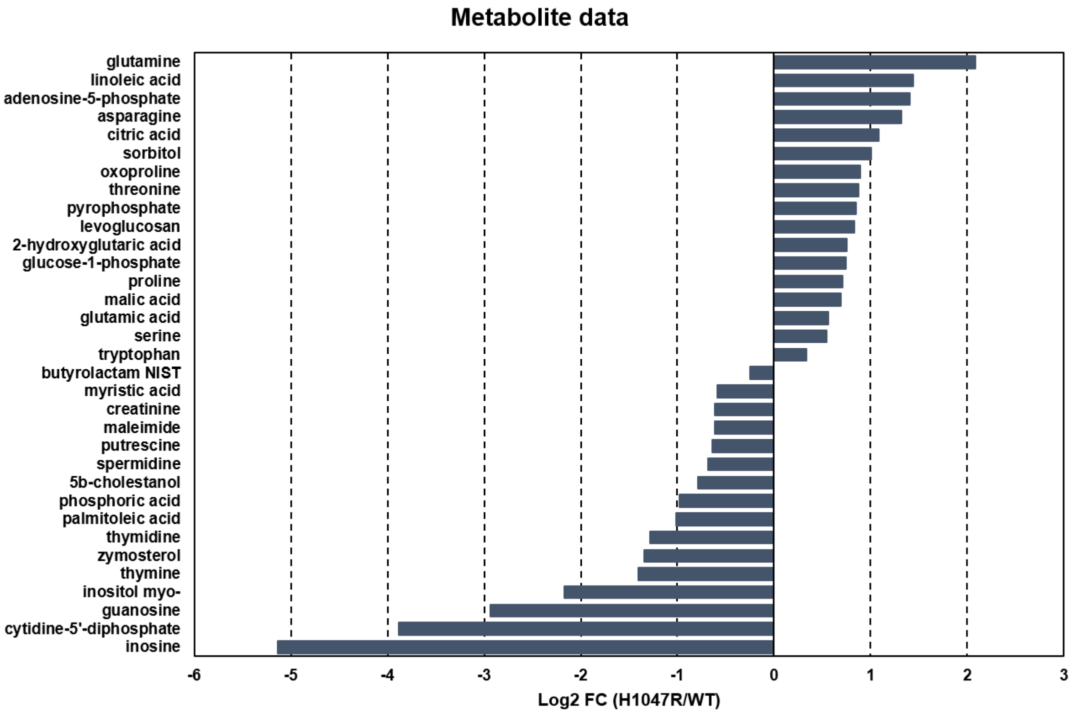

Figure S11. Comparison of metabolite levels based on available literature data. The data represent log 2 fold changes in metabolite levels comparing *PIK3CA H1047R* cells to wild type MFC10A cells; a positive fold change represents a metabolite was found higher in the mutant cells compared to wild type. All metabolites listed have $p < 0.05$ with negative binomial exact test. Data obtained from supplementary information of *The butterfly effect in cancer: A single base mutation can remodel the cell* [1].

TABLES

Table S1. Assignment and quantification of GC-MS detected metabolite features. The identities of the GC/MS features were confirmed either through running standards or matching to the NIST library. RI represents retention time indices. The data represent averages and SEM from seven biological replicate experiments and metabolites are ranked by the magnitudes of percentage changes. Pairwise Student's t-tests and false discovery rate (Benjamini and Hochberg procedure) analysis were used to evaluate statistical significance. FDR values and t test p values of < 0.05 are highlighted in red.

GC/MS profile: Effect of knock-in mutation on aqeuous metabolite abudnance in MCF10A

MS features	m/z	RI	% change	± error	T-test p value	FDR
dihydroxyacetone phosphate	484	2006	104%	26%	0.002	0.04
malic acid	419	1753	45%	10%	0.001	0.04
2 ketoglutaric acid	346	1652	44%	9%	0.007	0.06
3 phosphoglyceric acid	585	2254	43%	12%	0.022	0.07
fumaric acid	287	1449	40%	8%	0.013	0.06
phosphoenolpyruvic acid	453	1867	33%	22%	0.179	0.26
glutamic acid 432	432	1912	27%	7%	0.019	0.07
glutamic acid 330	330	1912	27%	7%	0.008	0.06
lactic acid 261	261	1154	12%	8%	0.151	0.24
asparagine	417	1945	12%	6%	0.154	0.24
lactic acid 233	233	1154	9%	9%	0.545	0.56
histidine	440	2220	-1%	8%	0.265	0.33
alanine 260	260	1195	-4%	7%	0.613	0.61
aspartic acid 418	418	1793	-5%	9%	0.458	0.48
aspartic acid 302	302	1793	-5%	9%	0.452	0.48
isoleucine	302	1379	-5%	5%	0.194	0.28
aspartic acid 390	390	1793	-5%	9%	0.415	0.46
alanine 232	232	1195	-6%	7%	0.304	0.35
citric acid 591	591	2223	-8%	8%	0.289	0.34
citric acid 459	459	2223	-8%	7%	0.289	0.34
glycine 246	246	1223	-8%	6%	0.049	0.13
glycine 218	218	1223	-9%	6%	0.052	0.13
cysteine	406	1845	-10%	8%	0.137	0.23
serine 302	302	1628	-11%	5%	0.091	0.17
serine 288	288	1628	-11%	5%	0.081	0.15
serine 390	390	1628	-12%	5%	0.122	0.21
threonine	404	1659	-14%	4%	0.007	0.06
glutamine	431	2061	-14%	5%	0.067	0.15
tyrosine	466	2269	-14%	4%	0.025	0.08
pyroglutamic acid	300	1596	-15%	3%	0.003	0.04
phenylalanine	336	1732	-15%	4%	0.019	0.07
valine	288	1310	-17%	6%	0.015	0.06
pyruvic acid	174	941	-19%	8%	0.061	0.14
methionine	320	1610	-21%	5%	0.014	0.06
lysine	431	2019	-22%	6%	0.051	0.13
glycerol	377	1522	-28%	13%	0.080	0.15
G3P+GPC (a)	571	2160	-43%	11%	0.235	0.30
G3P+GPC (b)	571	2205	-43%	12%	0.223	0.30

Table S2. Lipid metabolite ratios are altered in the PIK3CA mutant cells. Analysis was performed by GC-MS and data from both U-13C6 glucose U-13C5 glutamine tracer experiments were included. Both free fatty acids and fatty acid methyl esters were detected. Averages and S.E.M. from seven separate biological experiments are represented (apart from methyl stearate where S/N was low and data from only three independent biological replicates were included).

Effect of mutant PIK3CA on lipid metabolite abundance in MCF10A				
**lipid metabolite ratios**		**% change**	**error (SEM)**	**pairwise t test**
methyl palmitate, ratio to	methyl oleate	9%	8%	0.98
	methyl stearate	21%	7%	0.10
	oleic acid	17%	3%	0.01
	methyl linolenate	22%	7%	0.03
methyl oleate, ratio to	methyl palmitate	-5%	7%	0.22
	methyl stearate	-4%	12%	0.65
	oleic acid	11%	7%	0.10
	methyl linolenate	14%	5%	0.03
methyl stearate, ratio to	methyl palmitate	-17%	5%	0.09
	methyl oleate	8%	15%	0.73
	oleic acid	2%	10%	0.95
	methyl linolenate	14%	5%	0.07
methyl linolenate, ratio to	methyl palmitate	-16%	5%	0.03
	methyl oleate	-11%	4%	0.04
	methyl stearate	-12%	4%	0.12
	oleic acid	-2%	6%	0.72
oleic acid, ratio to	methyl palmitate	-14%	2%	0.03
	methyl oleate	-7%	6%	0.47
	methyl stearate	1%	9%	0.97
	methyl linolenate	5%	6%	0.42

Table S3. Extracellular consumption and release profile. The table above represents data from three independent biological replicate experiments.

Flux (fmol/cell/hr)	Parent		PIK3CA mutant		% change	t-test p value	
	Mean	SEM	Mean	SEM		two-tailed	paired
alanine ^{13}C	26	6	20	3	-25%	0.39	0.44
choline	-1.7	0.3	-1.8	0.2	3%	0.89	0.76
glucose ^{13}C	-1072	159	-1152	120	7%	0.71	0.49
glutamate (C4) ^{12}C	16	4	35	2	113%	0.02	0.08
glutamine	-52	26	-80	22	54%	0.47	0.04
lactate ^{12}C	109	25	80	14	-27%	0.37	0.16
lactate ^{13}C	1443	184	1661	194	15%	0.46	0.27
pyruvate ^{12}C	1.4	0.3	0.5	0.04	-61%	0.03	0.06
pyruvate ^{13}C	25	1	15	1	-40%	0.01	0.08

Table S4. ^{1}H NMR spectral resonance assignments for culture media samples

Assignment	ppm	moiety
alanine ^{13}C	1.36	$^{13}\mathbf{CH_3}CH(NH_2)\,COOH$
choline	3.19	$(C\mathbf{H_3})_3N(CH_2)_2OH$
glucose ^{13}C	5.08	Carbon-1 α anomeric H
glutamate (C4) ^{12}C	2.34	$HOOCCH(NH_2)CH_2C\mathbf{H_2}COOH$
glutamine	2.42	$HOOCCH(NH_2)CH_2C\mathbf{H_2}COH_2N$
lactate ^{12}C	1.33	$C\mathbf{H_3}CH(OH)COOH$
lactate ^{13}C	1.42	$^{13}C\mathbf{H_3}CH(OH)\,^{\mathbf{13}}COOH$
pyruvate ^{12}C	2.36	$C\mathbf{H_3}COCOOH$
pyruvate ^{13}C	2.47	$^{13}C\mathbf{H_3}COCOOH$

Table S5. ^{1}H NMR spectral resonance assignments for choline metabolites of cell extract samples

Assignment	ppm	moiety
choline	3.21	$(C\mathbf{H_3})_3N^+(CH_2)_2OH$
phosphocholine	3.22	$(C\mathbf{H_3})_3N^+(CH_2)_2OPO(OH)_2$
glycerophosphorylcholine	3.24	$(C\mathbf{H_3})_3N^+(CH_2)_2OPOO^-OCH_2CH(OH)CH_2(OH)$

Table S6. Fatty acid ISA modelled parameters. The table above represents data from three biological U-$^{13}C_6$ glucose culture replicates and four biological U-$^{13}C_5$ glutamine culture replicate experiments

methyl palmitate ISA parameters		MCF10A WT		PIK3CA mutant		Statistical significance	
		mean	SEM	mean	SEM	T test p value	
Labelled Acetyl CoA	Glucose labelled culture	0.55	0.049	0.60	0.008	0.435	
	Glutamine labelled culture	0.11	0.015	0.10	0.002	0.107	
De Novo Synthesis	Glucose labelled culture	0.26	0.055	0.36	0.009	0.152	
	Glutamine labelled culture	0.20	0.060	0.31	0.034	0.046	*

REFERENCES

1 Hart, J. R. *et al.* The butterfly effect in cancer: A single base mutation can remodel the cell. *Proceedings of the National Academy of Sciences of the United States of America* **112**, 1131-1136, DOI:10.1073/pnas.1424012112 (2015).
